# Intratumoral Convergence of the TCR Repertoires of Effector and Foxp3^+^ CD4^+^ T cells

**DOI:** 10.1371/journal.pone.0013623

**Published:** 2010-10-26

**Authors:** Michal Kuczma, Magdalena Kopij, Iwona Pawlikowska, Cong-Yi Wang, Grzegorz A. Rempala, Piotr Kraj

**Affiliations:** 1 Center for Biotechnology and Genomic Medicine, Medical College of Georgia, Augusta, Georgia, United States of America; 2 Department of Biostatistics and the Cancer Center, Medical College of Georgia, Augusta, Georgia, United States of America; New York University, United States of America

## Abstract

The presence of Foxp3^+^ regulatory CD4^+^ T cells in tumor lesions is considered one of the major causes of ineffective immune response in cancer. It is not clear whether intratumoral T_reg_ cells represent T_reg_ cells pre-existing in healthy mice, or arise from tumor-specific effector CD4^+^ T cells and thus representing adaptive T_reg_ cells. The generation of T_reg_ population in tumors could be further complicated by recent evidence showing that both in humans and mice the peripheral population of T_reg_ cells is heterogenous and consists of subsets which may differentially respond to tumor-derived antigens. We have studied T_reg_ cells in cancer in experimental mice that express naturally selected, polyclonal repertoire of CD4^+^ T cells and which preserve the heterogeneity of the T_reg_ population. The majority of T_reg_ cells present in healthy mice maintained a stable suppressor phenotype, expressed high level of Foxp3 and an exclusive set of TCRs not used by naive CD4^+^ T cells. A small T_reg_ subset, utilized TCRs shared with effector T cells and expressed a lower level of Foxp3. We show that response to tumor-derived antigens induced efficient clonal recruitment and expansion of antigen-specific effector and T_reg_ cells. However, the population of T_reg_ cells in tumors was dominated by cells expressing TCRs shared with effector CD4^+^ T cells. In contrast, T_reg_ cells expressing an exclusive set of TCRs, that dominate in healthy mice, accounted for only a small fraction of all T_reg_ cells in tumor lesions. Our results suggest that the T_reg_ repertoire in tumors is generated by conversion of effector CD4^+^ T cells or expansion of a minor subset of T_reg_ cells. In conclusion, successful cancer immunotherapy may depend on the ability to block upregulation of Foxp3 in effector CD4^+^ T cells and/or selectively inhibiting the expansion of a minor T_reg_ subset.

## Introduction

The observation that tumor antigen-specific B and T cells are activated in the course of tumor growth led to the presumption that augmenting the immune system function will lead to the eradication of tumor cells [1]. A multitude of cancer vaccines were designed to harness potent effector functions and exquisite specificity of the immune system to combat cancer. However, immunotherapy protocols used so far have had only a limited success what was attributed to poor recruitment of antigen specific T cells into tumor lesions, inadequate stimulation by antigens derived from tumor cells causing T cell anergy instead of T cell activation and, in particular, to the presence of regulatory T cells (T_reg_) expressing a transcription factor Foxp3 [2]–[4]. Despite their importance for cancer immunity, the origin of T_reg_ cells in tumors remains little known.

Foxp3^+^ T_reg_ cells are a specific population of CD4^+^ T lymphocytes that control normal immune homeostasis and self-tolerance [5]. T_reg_ cells were identified as the major obstacle to effective antitumor immunotherapy [6]–[8]. The abundance of these cells in peripheral blood is increased in patients with multiple types of cancer and their prevalence among tumor-infiltrating lymphocytes correlated with poor clinical prognosis [9]–[11]. In contrast, removal or inactivation of T_reg_ cells led to enhanced antitumor immune response and better efficacy of cancer vaccines [12]–[15].

Two major subsets of Foxp3^+^ T_reg_ cells, natural and adaptive T_reg_ cells, were defined based on whether their suppressor function is acquired during normal T cell development in the thymus or following TCR stimulation in peripheral tissues or *in vitro* [16], [17]. The lack of appropriate surface markers so far precluded the analysis of the contribution of these subsets to the peripheral pool of regulatory cells in healthy and tumor-bearing mice expressing diverse, polyclonal TCR repertoire. Though the suppressor function of the two T_reg_ subsets was found similar in *in vitro* tests, little is known how different are their homing properties, antigen specificities and the ability to expand *in vivo* in response to antigen stimulation and cytokines. The recent evidence that natural and adaptive T_reg_ subsets have different gene expression signature and synergize to establish peripheral tolerance suggests that they serve non-redundant functions [18], [19]. In a recent report we have shown that the level of Foxp3 expression and TCR repertoires define two subsets of T_reg_ cells in peripheral lymphoid organs [20]. The dominant fraction of peripheral T_reg_ cells consistently expresses high level of Foxp3 and a characteristic set of TCRs not utilized by naive effector CD4^+^ T cells. The second T_reg_ subset expressing low level of Foxp3, CD25 and GITR and constituting only a small fraction of T_reg_ cells, could up- or downregulate Foxp3 when stimulated with antigen and utilized TCRs shared with naive T cells. The modulation of Foxp3 expression was dependent on the presence of cytokines, especially TGF-β, that increased the fraction of cells upregulating Foxp3. This T_reg_ subset was able to efficiently expand in lymphopenic mice and in mice undergoing immune response to antigen where it became a major population of antigen-specific T_reg_ cells. Since TCR repertoires expressed by naive and T_reg_ CD4^+^ T cells show only a minimal overlap, we postulate that T_reg_ subset expressing TCRs shared with naive cells represents adaptive T_reg_ cells [20]–[22]. Our findings corroborated recent report showing that two subsets of peripheral T_reg_ cells exist in mice not subject to any deliberate antigen stimulation [23].

The heterogeneity of T_reg_ population was also described in humans. Two functional subsets of Foxp3^+^ T_reg_ cells with distinct capacity to secrete IL-10 and TGF-β and using different mechanisms of suppression were defined by expression of ICOS [24]. High levels of ICOS was also expressed on melanoma tumor infiltrating T_reg_ cells producing high level of IL-10 [25]. In another report the level of Foxp3 expression and CD45RA defined populations of resting (CD45RA^+^Foxp3^lo^) and activated (CD45RA^-^Foxp3^hi^) T_reg_ cells and a population of cells (CD45RA^-^Foxp3^lo^) able to produce IL-2 and IFN-γ [26]. Resting T_reg_ cells convert into activated T_reg_ cells and the relative proportions of these subsets change in aged and diseased individuals. In summary, both in humans and in mice peripheral T_reg_ cells are heterogenous and include subsets with different phenotypic and functional characteristics that are subject to dynamic regulation. It is not known what is the contribution of the recently identified T_reg_ subsets to the generation of T_reg_ population in cancer, especially to the population of T_reg_ cells in tumor lesions.

While it is well established that upregulation of Foxp3 in effector CD4^+^ T cells leads to the generation of T_reg_ cells, it is unclear what is the contribution of converted and pre-existing T_reg_ cells to the population of Foxp3^+^ T cells in tumors [27]. Experiments investigating T_reg_ cells in tumor-bearing mice that used adoptively transferred effector and T_reg_ cells expressing transgenic TCR specific for tumor antigen have found that both subsets contribute to T_reg_ population [28]. However, it is not known how the number of transferred cells affects the recruitment of antigen-specific effector and T_reg_ CD4^+^ T cells into tumor draining lymph nodes and tumors and if their migration, sensitivity to cytokines and/or conversion into T_reg_ cells is the same as CD4^+^ T cells that naturally developed in recipient mice. Moreover, since the majority of T_reg_ cells express different TCRs than effector T cells, it is unclear if T_reg_ cells expressing the same transgenic TCR as effector T cells used in these studies are equivalent to the majority of T_reg_ cells present in healthy mice.

To determine the origin of T_reg_ cells in cancer, we used an experimental model in which immune response, of both effector and T_reg_ cells, to tumor-associated antigens can be characterized in mice with a restricted and readily characterized CD4^+^ T cell repertoire. These mice undergo natural generation of a polyclonal CD4^+^ T cell repertoire with the set of TCR sequences that are used by the majority of natural T_reg_ cells distinct and readily differentiable from the set of sequences used by effector T cells [22]. This allows us to ask whether tumor-associated T_reg_ cells arise from the repertoire of TCRs used by natural T_reg_ cells or from the repertoire used by effector cells. We show that T_reg_ population in tumors is dominated by T cells expressing the same TCRs as effector T cells. These data suggest that T_reg_ in tumors are generated by expansion of a minor subset of T_reg_ cells that shares TCRs with effector T cells or by conversion of effector CD4^+^ T cells and thus could represent adaptive T_reg_ cells. In contrast, we found that pre-existing T_reg_ cells, expressing an exclusive set of TCRs, that dominate in healthy mice constitute only a small proportion of T_reg_ cells in tumors. We also demonstrate that immunotherapy aimed at stimulating immune response does not change proportions of T_reg_ and effector CD4^+^ T cells in tumor-bearing mice.

## Methods

### Ethics Statement

Full details of the study and all procedures performed on animals were approved by the Institutional Animal Care and Use Committee of the Medical College of Georgia (approval number 09-06-213) and complied with all state, federal, and NIH regulations.

### Mice

TCR^mini^-Foxp3^GFP^ mice were generated by crossing TCR^mini^ mice expressing restricted TCR repertoire with transgenic mice expressing Foxp3^GFP^ reporter transgene [20], [22]. TCR^mini^ mice harbor a mini-repertoire of TCR α chains encoded by Vα2.9 and Jα26 (or Jα2) associated with one rearranged TCR Vβ14 chain. The mice were crossed to TCRα chain knockout mice to prevent rearrangements of endogenous TCRα chains. Thus, the diversity of the TCR repertoire depends on the CDR3 region of the TCRα chain. Mice expressing a congenic marker Ly5.1 were purchased form Jackson Laboratory (Bar Harbor, ME) and crossed with transgenic Foxp3^GFP^ mice. All animals used were on the C57BL6 background. Mice were maintained in a specific pathogen-free conditions in a controlled environment which included filtered air and a 12 hour light/dark cycle. All animals had free access to food and water.

### Tumors

The mouse melanoma cell line B16F1 was obtained from ATCC. For most experiments B16 melanoma expressing Ep63K peptide as part of the influenza virus nucleoprotein was used. A DNA fragment encoding Ep63K peptide (17 amino-acids long) was introduced between amino acids 264–280 of the nucleoprotein (gift of Dr. G. Price) using standard molecular biology techniques. Modified nucleoprotein (after removal of a stop codon) was subcloned into pEGFP-N1 vector (EcoRI-SalI sites, SalI site within Ep63K peptide was mutated during cloning process) to tag it with green fluorescent protein (GFP) at the C terminus and then cloned into LZRS-pBMN-Z retroviral vector (between EcoRI and NotI sites). Retroviral particles were generated as described by transfecting the Phoenix 293T packaging cell line, a human embryonic kidney line that produces helper-free ecotropic retroviruses (provided by Dr. G. Nolan). Retrovirally transduced cells expressed NP-Ep63K-GFP complexes for at least three weeks of *in vitro* culture as assessed by stable GFP expression. To produce tumors, B16 melanoma cells devoid of or expressing NP-Ep63K-GFP were sorted and expanded *in vitro* for 2–3 days before injection. Tumor cells (5×10^4^) were injected s.c. in the upper, inside of both thighs of 6–8 week old TCR^mini^-Foxp3^GFP^ mice and mice were analyzed after indicated time. Lymph nodes from two mice were pooled for TCR repertoire analyzes.

### Cell purification, flow cytometry and cell sorting

Single-cell suspensions were prepared from lymph nodes by mechanical disruption and cells were stained with antibodies available commercially (eBioscience or BD Biosciences). Tumor infiltrating lymphocytes (TILs) were prepared from tumor lesions by scrubbing tumor tissue into PBS with 0.1 M EDTA. B16 cell suspension (10^7^ cells/ml) was then overlaid on 5 ml of Lympholyte-M (Cedarlane, NC) gradient and spun at 2300×g for 20 min. at 24°C. The cells at the interphase were collected and, after washing with HBSS, stained with monoclonal antibodies for flow cytometry analysis and sorting. Cells were analyzed using FACSCanto flow cytometer (Becton Dickinson) and FACSDiva or WinList software. Cells were sorted on a MoFlo cell sorter (Cytomation). Purity of sorted populations exceeded 98.5%.

### Proliferation assay

Proliferation assays were performed with the population of total CD4^+^ T cells or CD4^+^Foxp3^GFP-^ cells sorted from B16 tumors and tumor-draining lymph nodes. TCR^mini^-Foxp3^GFP^ mice were inoculated with B16 melanoma expressing NP-Ep63K-GFP construct (5×10^4^ injected s.c.) and tumors were allowed to grow for 2 weeks. Sorted cells (10^5^/well) were incubated on a 96-well plate with irradiated splenocytes devoid of T cells (10^5^/well, 3000 Rad) and soluble anti-CD3ε (5 µg/ml). Proliferation responses were measured by adding 1 µCi/well of ^3^H-thymidine on day 3 of a 4-day culture.

### Two-Dimensional, Fluorescent, Single-Stranded Conformational Polymorphism Analysis of Vα2 Repertoires (2D-F-SSCP)

Analysis of the TCR repertoire using 2D-F-SSCP analysis was performed on sorted populations of effector and T_reg_ cells from control, tumor draining lymph nodes and tumor tissues of TCR^mini^-Foxp3^GFP^ mice injected with melanoma cells as described previously [22]. Tumor cells were injected s.c. in both thighs. For analysis brachial and axillary lymph nodes were considered control lymph nodes, inguinal lymph nodes were tumor draining lymph nodes. The purity of the sorted populations exceeded 98%. cDNA prepared from at least 10^5^ sorted cells was used for amplification of the TCRα chains. Fluorescent images were acquired by scanning the slab gel in a Typhoon 9410 imager (Amersham-Pharmacia) and analyzed with Image Master 5.0 Platinum software (Amersham-Pharmacia). DNA gels representing individual cell populations were aligned using control spots of known DNA sequence added to each sample. DNA for control spots was selected such that control spots fall outside the gel area utilized by sample DNA. Spot detection and spot comparison was accomplished automatically by image analysis software. In a few cases spots were manually aligned.

### Single-cell RT-PCR and TCR sequencing

The populations of naive CD44^−^CD62L^+^Foxp3^GFP-^, activated/memory CD44^+^CD62L^−^Foxp3^GFP-^ and Foxp3^GFPlo^ and Foxp3^GFPhi^ (or all Foxp3^GFP+^) T_reg_ CD4^+^ cells were sorted from cell suspensions prepared from control and draining lymph nodes and tumor infiltrate. For some experiments CD4^+^CD25^+^ T cells were sorted from control, draining lymph nodes and tumors. The purity of the sorted populations exceeded 98%. These populations were subsequently subjected to single-cell sorting as described previously [20]. Cells from two TCR^mini^-Foxp3^GFP^ mice were combined for cell sorting. We analyzed 316, 135, 135 TCRs from naive, activated and T_reg_ cells from control lymph nodes and 187, 176 and 136 TCRs from the respective populations isolated from draining lymph nodes. For tumors, 173 and 275 TCRs were analyzed from activated and T_reg_ cells. DNA sequencing was done in the DNA sequencing core facility at the University of Illinois.

### Cytokine and transcription factor detection by RT-PCR

CD4^+^Foxp3^GFP-^ and CD4^+^Foxp3^GFP+^ T cells were sorted from tumors and RNA was isolated with an RNeasy Mini Kit (Qiagen) and reverse transcribed using a Superscript kit (Invitrogen) according to the manufacturer's instructions. β-actin was used to normalize cDNA quantities and was amplified with the sense primer 5′CCTTCTACAATGAGCTGCGTGTGGC3′ and antisense primer 5′CATGAGGTAGTCTGTCAGGTCC3′. Cytokine and Foxp3 cDNA was amplified using the following primers: Foxp3 sense: 5′ATCCAGCCTGCCTCTGACAAGAACC3′, antisense: 5′GGGTTGTCCAGTGGACGCACTTGGAGC3′. These primers distinguish between amplification product of the endogenous Foxp3 gene (401 bp) and the transgenic transcript (1357 bp). IL-10, sense: 5′AGTGGAGCAGGTGAAGAGTG3′, antisense: 5′TTCGGAGAGAGGTACAAACG3′, TGF-β sense: 5′GCTACCATGCCAACTTCTGT3′, antisense: 5′CGTAGTAGACGATGGGCAGT3′.

### Proliferation inhibition assay

Suppressor function of Foxp3^GFP+^ T cells isolated from tumor-bearing TCR^mini^-Foxp3^GFP^ mice was investigated in the proliferation inhibition assay. CD4^+^Foxp3^GFP-^ cells (3×10^4^/well) were incubated on a 96-well plate with irradiated splenocytes (5×10^4^/well, 3000 Rad) and soluble anti-CD3ε (5 µg/ml). CD4^+^Foxp3^GFP+^ cells (2×10^4^/well) were sorted from tumor draining lymph nodes or tumors of TCR^mini^-Foxp3^GFP^ mice, inoculated two weeks earlier with B16 melanoma tumors, were added. Cells were sorted using MoFlo sorter, purity of sorted cells exceeded 98.5%. After 3 day culture proliferation was measured by adding 1 µCi/well of ^3^H-thymidine.

### Adaptive cell transfer

Donor cells for adaptive transfer were isolated by flow cytometry sorting of CD4^+^Foxp3^GFP-^ cells from Ly5.1^+/−^Foxp3^GFP^ mice or CD4^+^Foxp3^GFPhi^ cells from Ly5.1^+/+^Foxp3^GFP^ mice expressing wild-type TCR repertoires. CD4^+^Foxp3^GFP-^ (2×10^6^/mouse) and CD4^+^Foxp3^GFPhi^ cells (3×10^5^/mouse) were mixed and co-injected i.v. into recipient Ly5.1^-/-^ TCR^mini^ mice. TCR^mini^ mice were inoculated with B16 melanoma cells (10^5^/mouse s.c.) three days before cell transfer. Recipient mice were sacrificed 12 days after cell transfer and cell populations from lymph nodes and tumors were analyzed by flow cytometry.

### Production and immunization with bone marrow-derived dendritic cells expressing covalent A^b^Ep63K complex

Bone marrow was isolated from mice expressing A^b^Ep63K complex and lacking endogenous wild type A^b^ molecules and invariant chain [29]. Bone marrow depleted of erythrocytes was incubated with granulocyte-macrophage colony stimulating factor (GM-CSF), and IL-4 (50 units/ml). After 6–7 day culture bone marrow cells were stained for expression of A^b^, CD11c and B7-1. Cells positive for A^b^ were sorted with magnetic beads (Miltenyi Biotech) (purity of sorted cells >98%) and used for immunotherapy of tumor-bearing C57BL6 Foxp3^GFP^ mice. C57BL6 Foxp3^GFP^ mice (expressing wild type TCR repertoire) were inoculated s.c. with 5×10^4^ B16 cells expressing NP-Ep63K. 5×10^4^ bone marrow-derived dendritic cells expressing covalently bound complex of A^b^Ep63K were co-injected s.c. in the same site at the time of tumor inoculation. Injections of dendritic cells continued daily until mice were sacrificed.

### Statistical analysis

Diversity of the TCR repertoires was calculated using the Chao mean estimators of unobserved species with 95% confidence intervals obtained via bootstrap method based on 10,000 resamples [30]. To assess the similarity between the TCR repertoires we have calculated the relative entropy (or Kullback-Leibler distance) for each of the cell subsets against the pooled “superpopulation” consisting of all receptors and pooled relative frequencies [31]. For a given population repertoire the Kullback-Leibler distance against the superpopulation was calculated as the sum of the products between *p_k_* and *log(p_k_/q_k_)* where *p_k_* denotes the population frequency of the *k-*th TCR and *q_k_* denotes the pooled frequency of the *k*-th TCR (i.e., frequency in the superpopulation). The Kullback-Leibler distance from the superpopulation was then bias-corrected by subtracting the estimate of the first order bias given by *(Ĵ* *−1)/(2n)* where *Ĵ* is the Chao mean and *n* is the number of all cells in the given population. The confidence intervals for the bias-corrected Kullback-Leibler distances against superpopulation were calculated using the bootstrap method with 10,000 resamples. All calculation were done using R software package.

## Results

### B16 melanoma expressing Ep63K peptide as a tumor-associated antigen

**Figure 1:**
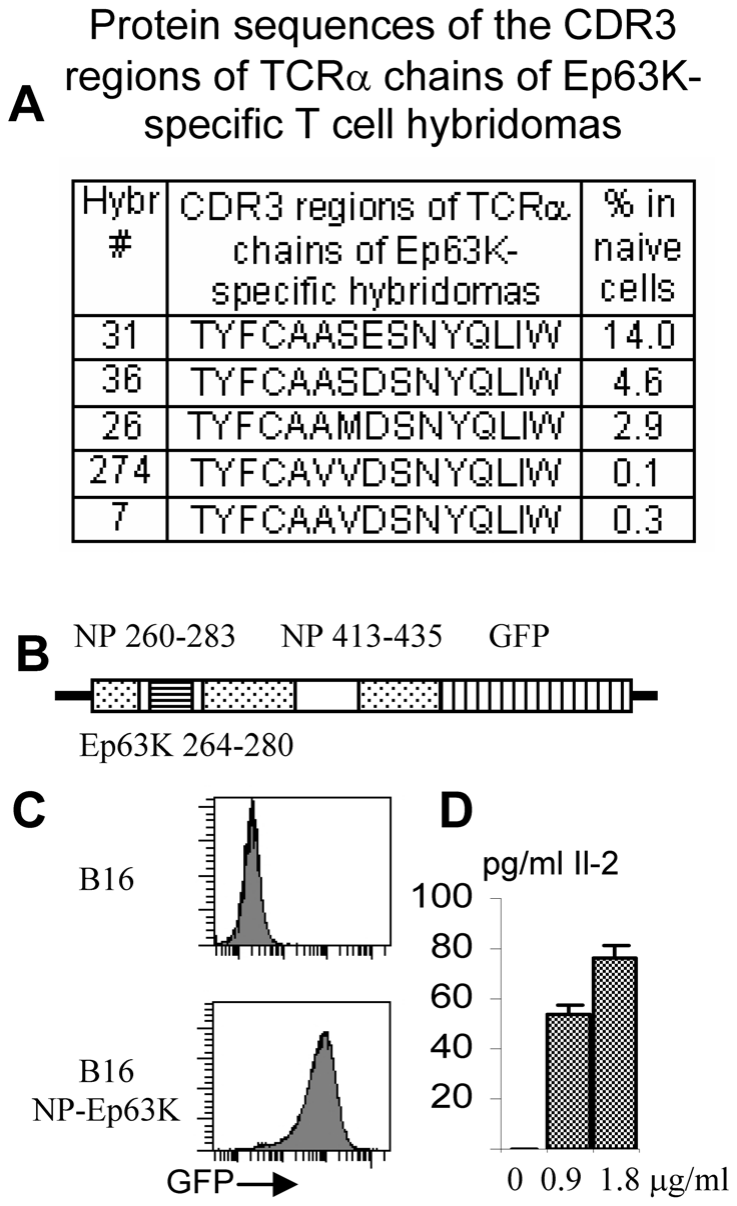
Amino-acid sequences of TCRα chain CDR3 regions of Ep63K-specific CD4^+^ T cell hybridomas obtained from TCR^mini^-Foxp3^GFP^ mice and generation of B16 tumors expressing tumor associated neo-antigen NP-Ep63K. (A) Amino-acid sequences of TCRα chain CDR3 regions of Ep63K-specific CD4^+^ T cell hybridomas obtained from TCR^mini^-Foxp3^GFP^ mice. (B) Production of the nucleoprotein-Ep63K-GFP expression construct. Sections encoding nucleoprotein are shown as dotted rectangles and GFP is shown as vertical lines. Parts of the nucleoprotein encoding peptides binding A^b^ are shown as clear rectangles and Ep63K peptide is shown as horizontal lines. Numbers show the range of amino acids constituting peptides binding to A^b^. (C) Flow cytometry analysis of the B16 melanoma cells expressing nucleoprotein-Ep63K-GFP. B16 melanoma cells were transduced with the LZRS-pBMN-Z retroviral vector expressing nucleoprotein-Ep63K-GFP fusion protein (lower histogram) or a control vector (upper histogram). (D) Recombinant nucleoprotein-Ep63K is processed by bone marrow derived dendritic cells and Ep63K peptide is recognized by specific CD4^+^ hybridoma 123.3. IL-2 (pg/ml) production in the supernatant of dendritic cells cultured without and with 0.9 or 1.8 µg/ml of the recombinant protein.

We have studied immune response in cancer in TCR^mini^-Foxp3^GFP^ mice using B16 transplantable tumor model [20]. TCR^mini^ mice express a naturally generated, polyclonal TCR repertoire where we can follow the frequency of individual T cell clones in cell subsets defined by expression of the Foxp3 and surface markers [20], [22]. The relative diversities and the size of individual clones of CD4^+^ T cell subsets in TCR^mini^ mice are similar to the natural TCR repertoire as recently determined by high throughput sequencing of TCRs expressed by human T cells [32]. The Ep63K peptide is a cognate antigen recognized by the rearranged TCR that was used to produce TCR^mini^ mice. The TCRs specific for Ep63K were identified and their frequency is known in unmanipulated TCR^mini^-Foxp3^GFP^ mice and in mice undergoing response to antigen following immunization with peptide and CFA (Fig. 1A) [20], [33]. To take advantage of the defined TCR repertoire of Ep63K-specific T cells, B16 melanoma was modified to express this peptide. Since it is generally accepted that tumor tolerance and immunity are induced by cross-presentation of tumor antigens, even for class II MHC positive tumors, we have prepared B16 cells expressing Ep63K peptide as part of the influenza virus nucleoprotein (NP)(Fig. 1B) [34]. During viral infection, NP expressed in cells lacking MHC class II molecules, is processed to peptides p260–283 and p413–435, which bind A^b^ on antigen presenting cells [35]. A DNA fragment encoding Ep63K peptide (17 amino-acids long) was introduced between amino acids 264–280 of the nucleoprotein using standard molecular biology techniques. The amino acids flanking mutant peptide p260–283 were preserved in the modified nucleoprotein to ensure proper proteolytic processing of NP-Ep63K. Modified protein was tagged with green fluorescent protein and expressed in B16 melanoma (Fig. 1C). Cross-presentation and processing of modified nucleoprotein was tested by stimulating Ep63K-specific hybridoma 123.3 with recombinant NP-Ep63K (Fig. 1D)[36].

Ep63K peptide generated by processing of modified NP represents antigenic epitope for which immune system is not tolerant. This class of tumor-associated antigens could potentially induce more robust immune response than antigenic epitopes generated by processing of natural, unmutated self proteins and offers best potential for successful immunotherapy. Recent reports show remarkable accumulation of mutations by tumor-associated antigens, including proteins that contribute to the neoplastic process [37]. Mutant proteins are a source of novel and unique antigenic epitopes and demonstrate that tumor cells themselves are a source of a polyvalent vaccine [38]. These new findings suggest that appropriate manipulation of the immune system could offer an opportunity to exploit the immunogenicity of the tumors for cancer immunotherapy.

### Analysis of the TCR repertoire in TCR^mini^-Foxp3^GFP^ mice bearing late stage melanoma tumors reveals expansion of antigen-specific effector and regulatory CD4^+^ T cells

**Figure 2:**
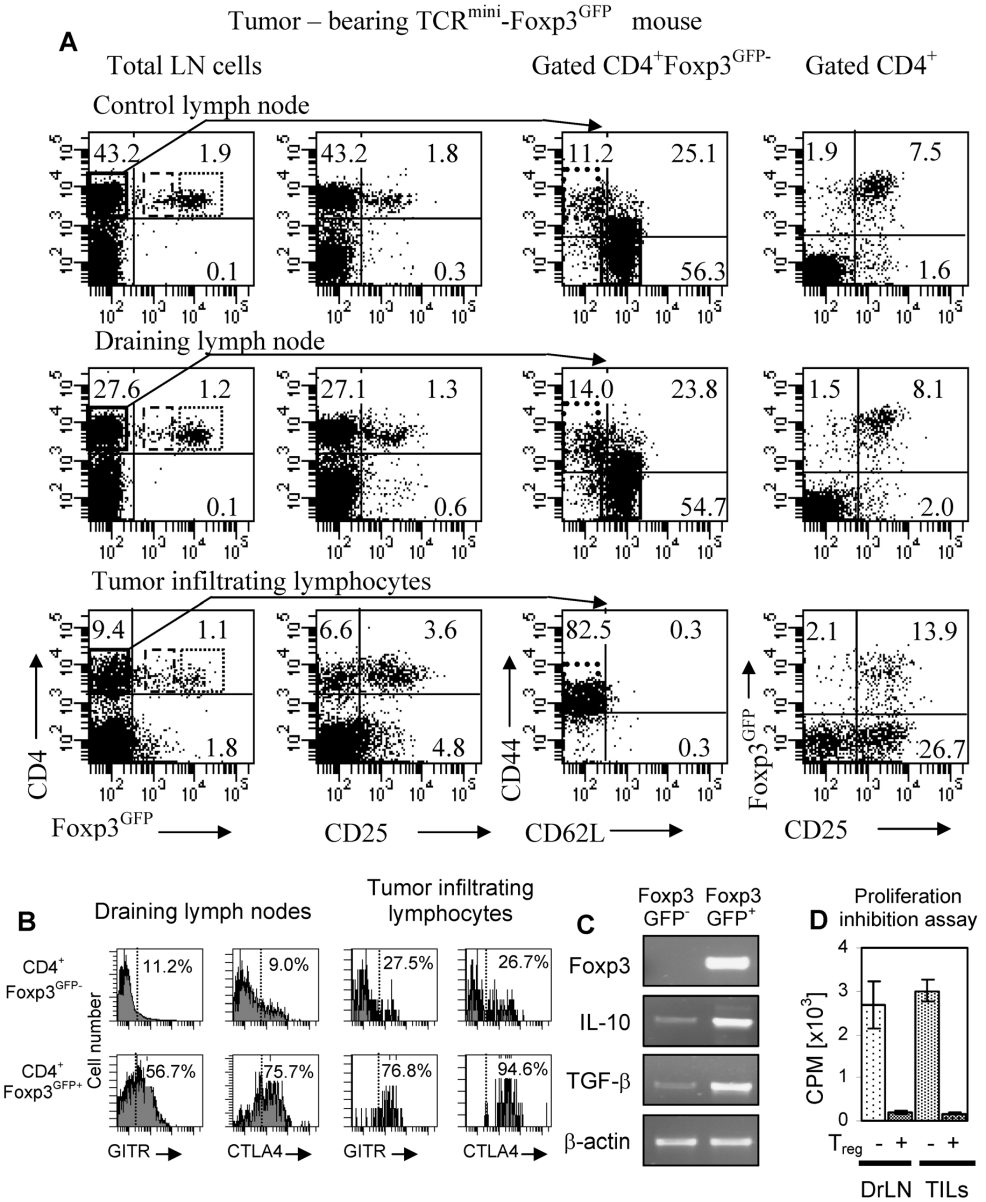
Flow cytometry, gene expression and functional analysis of effector and T_reg_ cells in TCR^mini^-Foxp3^GFP^ mice bearing B16 melanoma tumors expressing NP-Ep63K. (A) Cells from control, draining lymph nodes and tumor infiltrates were isolated and single cell suspensions were stained with the relevant antibodies. Expression of Foxp3^GFP^ (left column) and CD25 (second column) on CD4^+^ T cells are shown. Expression of activation markers CD44 and CD62L is shown on gated effector CD4^+^ Foxp3^−^ cells (third column) and Foxp3 and CD25 expression are shown on gated CD4^+^ T cells (right column). Gates used to define Foxp3^GFP-^ cells (left column, continuous line) and subsets of naive CD44^−^CD62L^+^ (third column, continuous line) and activated CD44^+^CD62L^−^ (third column, dotted line) cells as well as Foxp3^GFPlo^ (left column, broken line) and Foxp3^GFPhi^ (left column, dotted line) T_reg_ cells for TCR repertoire studies are shown as rectangles. Naive cells were absent in tumors. Figure shows representative data of at least five mice analyzed. (B) Expression of GITR (left panels) and CTLA-4 (right panels) in CD4^+^Foxp3^GFP-^ (upper panels) and CD4^+^Foxp3^GFP+^ (lower panels) cells isolated from tumor draining lymph nodes and tumors. Two individual mice were analyzed. (C) Analysis of Foxp3, IL-10 and TGF-β expression in Foxp3^GFP-^ and Foxp3^GFP+^ CD4^+^ T cells isolated from tumors. Sorted cells were lysed directly and gene expression was detected by RT-PCR. Samples were normalized for β-actin expression. Two individual mice were analyzed. (D) Foxp3^GFP+^CD4^+^ T cells isolated from the draining lymph nodes (DrLN) or tumors (TILs) suppress proliferation of effector CD4^+^ T cells. One of two experiments is shown.

To investigate how populations of CD4^+^ effector and T_reg_ cells change during immune response in cancer and how these two T cell subsets contribute to the T cell population in the tumor-draining lymph nodes and tumor tissue, we have followed immune response to transplantable B16 melanoma. T_reg_ cells are known to promote growth of B16 melanoma by inhibiting immune responses to melanoma-associated antigens [12]. B16 melanoma expressing NP-Ep63K was inoculated into TCR^mini^-Foxp3^GFP^ mice and CD4^+^ T cell populations were analyzed in the control and tumor-draining lymph nodes and tumors 19 days after tumor inoculation. Flow cytometry analysis shows increased proportion of activated cells in the draining lymph nodes and tumors consistent with the ongoing immune response (Fig. 2A). Increased proportion of activated T cells is however paralleled by the concomitant increase of the Foxp3^+^ T_reg_ cells. CD4^+^Foxp3^GFP+^ cells in the tumor draining lymph nodes and tumors express higher levels of CTLA-4 and GITR than activated Foxp3^GFP-^ cells (Fig. 2B). These cells express Foxp3 and high levels of IL-10 and TGF-β, cytokines known to contribute to the suppressor function of T_reg_ cells (Fig. 2C). Foxp3^GFP+^ cells sorted from B16 tumors are able to suppress proliferation of effector CD4^+^ T cells *in vitro* (Fig. 2D). In conclusion, phenotypic and functional analyzes strongly suggest that CD4^+^Foxp3^GFP+^ T cells that develop in melanoma tumors represent genuine T_reg_ cells and not cells that transiently upregulate Foxp3 without acquiring suppressor function [39].

**Figure 3:**
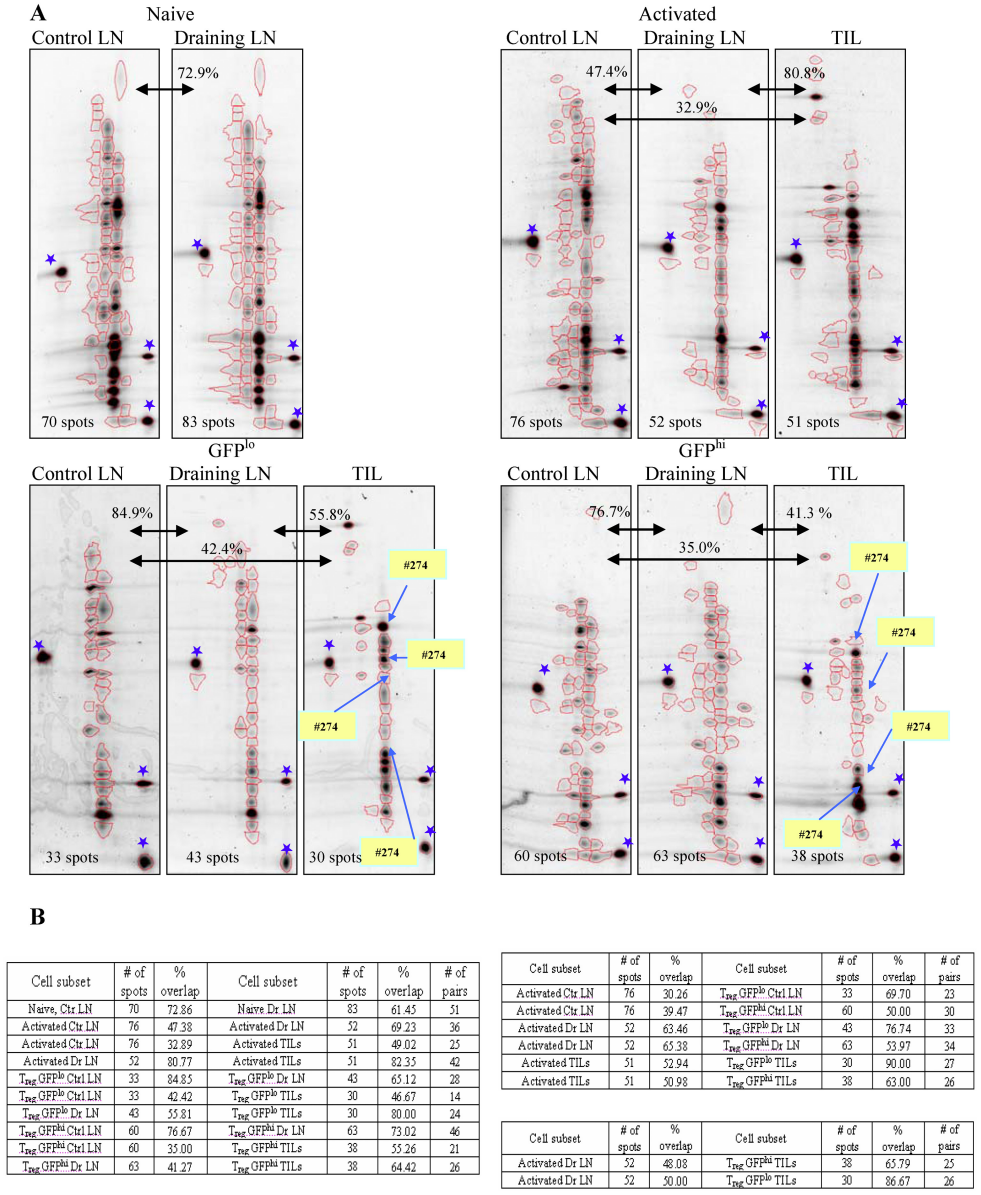
Analysis of TCR repertoires using 2D-F-SSCP. (A) Spot patterns representing TCR repertoires of naive, activated effector CD4^+^ T cells and Foxp3^GFPlo^ and Foxp3^GFPhi^ T_reg_ cells isolated from control and draining lymph nodes and tumors of tumor-bearing TCR^mini^-Foxp3^GFP^ mice. Blue asterisks indicate control spots used to overlay gel images for analysis. Spots representing T cells expressing the same TCR as Ep63K-specific hybridoma #274 are shown in the populations of Foxp3^GFPlo^ and Foxp3^GFPhi^ cells in tumors. The number of spots defined on each gel by image analysis software is shown in lower left corner of each gel image. Similarity between gels is shown as percentage of overlapping spots. Gels compared are indicated by arrows. (B) Pair wise comparison of the total number and number of overlapping spots between naive, activated and T_reg_ CD4^+^ T cell populations isolated from control and draining lymph nodes and tumors of tumor-bearing TCR^mini^-Foxp3^GFP^ mice. Left table includes comparison of cell populations isolated from different anatomical locations, right upper table shows comparisons of cell populations isolated from the same organ and right lower table shows comparisons of different cell populations isolated from different organs.

To determine the diversity of CD4^+^ T cell subsets and assess the abundance of individual T cell clones, we have used two-dimensional, fluorescent, single-stranded conformational polymorphism (2D-F-SSCP) analysis [22]. TCRα chains were amplified from naive CD44^−^CD62L^+^Foxp3^GFP-^, activated/memory CD44^+^CD62L^−^Foxp3^GFP-^ and Foxp3^GFPlo^ and Foxp3^GFPhi^ T_reg_ CD4^+^ cells sorted from control and draining lymph nodes and tumor infiltrate (Fig. 3). We have divided T_reg_ population into cells expressing high and low level of the Foxp3^GFP^ reporter since they constitute functionally different T_reg_ subsets in unmanipulated mice [20]. Fluorescent PCR products of TCRα chains were first separated according to their length and subsequently according to their nucleotide sequence. DNA gels representing individual cell populations were aligned using control spots and pair wise comparison of gel images was accomplished by analysis software. The similarity of TCR repertoires was assessed by counting the numbers of overlapping DNA spots. As shown in Fig. 3, the gels representing the TCR repertoires of naive cells in the control and the draining lymph nodes are very similar (72.9% of overlapping spots) since T cell clones in these subsets are not subject to antigen-driven selection and expansion. In contrast, the TCR repertoires of activated and Foxp3^GFPlo^ and Foxp3^GFPhi^ T_reg_ cells show various degree of similarity between anatomical locations. Analysis of the overlapping spots shows that populations of activated cells in the draining lymph nodes and tumors are very similar while they differ from the corresponding population in the control lymph nodes. Similar relationship was observed for Foxp3^GFPlo^ and Foxp3^GFPhi^ subsets. The TCR repertoires of Foxp3^GFPlo^ and Foxp3^GFPhi^ cells from tumors were more similar to the repertoires of the respective T_reg_ populations in the draining lymph nodes than in control lymph nodes. In addition, the TCR repertoires of activated T cells and Foxp3^GFPlo^ and Foxp3^GFPhi^ subsets are more similar in tumor draining lymph nodes and tumors than in the control lymph nodes. This suggests that T cell clones from effector cells are recruited into T_reg_ population in tumor draining lymph nodes and tumors. To confirm that clones expanded in T_reg_ populations express the same TCRs as activated T cells, we have isolated and sequenced spot DNA. The spots corresponding to the most prominent Ep63K-specific clone are shown on the gel image representing a population of T_reg_ cells in tumor tissue (Fig. 3A). In summary, analysis of gel images reveals changes of TCR repertoires characteristic of the ongoing clonal selection and expansion and suggests that immune response to tumor-derived antigens resembles response to conventional antigen stimulation [40].

### Tumor antigen-specific T cell clones expressing the same TCRs are concurrently expanded in effector and T_reg_ subsets in the draining lymph nodes and tumor tissue

**Figure 4:**
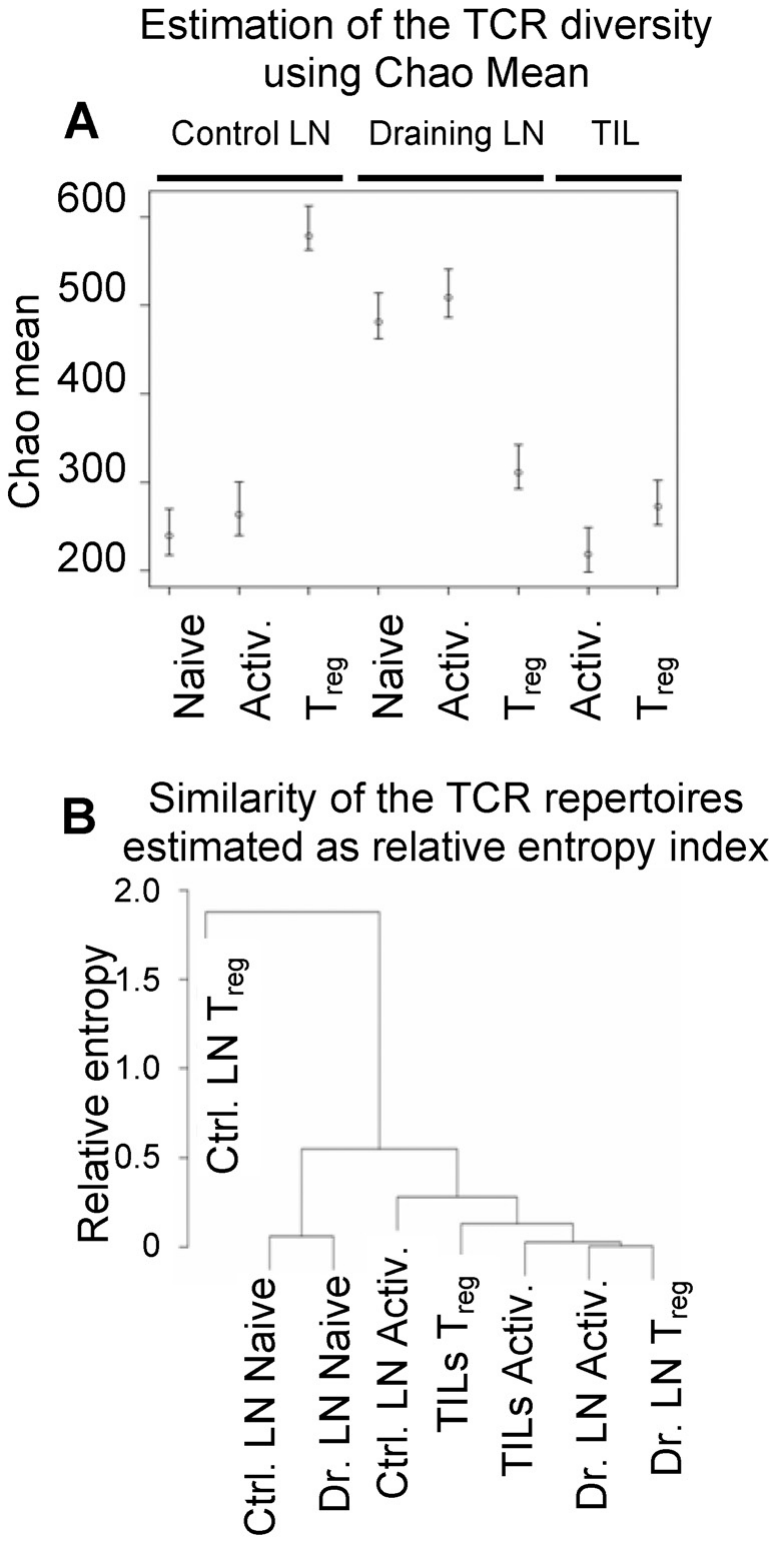
Analysis of the TCR repertoires of naive, activated and T_reg_ cells in control and draining lymph nodes and tumor lesions of tumor-bearing TCR^mini^-Foxp3^GFP^ mice. (A) Estimation of the TCR repertoire diversity using Chao mean. The data range spanned by vertical lines represent 95% confidence interval for Chao mean. The circles represent values of Chao estimator. (B) Estimation of the similarities of the TCR repertoires presented as a dendrogram based on the differences of the relative entropy against the pooled population for TCRs expressed by naive, activated (Activ.) and T_reg_ cells isolated from control (Ctrl. LN) and draining (Dr. LN) lymph nodes and tumor infiltrate (TILs). The dendrogram construction begins with each cell subset being a separate cluster. Then, the most similar cell populations (with the smallest difference in their relative entropies) are joined. We continue the process until we obtain a single cluster. The distance between two clusters is taken as the maximum difference in relative entropies of their members.

Analysis of TCR repertoires in mice bearing advanced tumors shows extensive changes of effector and T_reg_ cells isolated from various anatomical locations. Populations of activated and T_reg_ cells have been reshaped as evidenced by clonal expansions and contractions. The most significant changes affected populations of Foxp3^GFPlo^ and Foxp3^GFPhi^ cells in tumors and included a significant contribution of Ep63K-specific T cells expressing the same TCRs as activated effector cells. To further understand and characterize immune response in cancer, we sought to determine in what anatomical location and at what stage of the tumor growth the cellular changes that underlie the failure of immune response in cancer first occur. We analyzed TCR^mini^-Foxp3^GFP^ mice bearing early stage, 10 day old B16 tumors. This was the earliest time when we could isolate sufficient number of cells from tumors. TCR repertoires were analyzed by sorting single CD4^+^ T cells from populations of naive, activated and Foxp3^GFP+^ T_reg_ cells from control and draining lymph nodes and TILs and by sequencing TCRα chain genes [20], [22]. Naive population was not sorted from tumor tissue since almost all cells expressed an activated phenotype. The diversity of TCRs within populations and the overlap between populations were assessed using Chao mean and relative entropy index respectively (Fig. 4A, B)[20], [22]. The TCR diversity of naive and activated CD4^+^ T cells increased in tumor draining lymph nodes in comparison to control lymph nodes and decreased in tumor tissue. Increased diversity in the draining lymph nodes may reflect recruitment and expansion of T cell clones associated with inflammatory response in the neighboring tissues. Further clonal expansion and selection occurred in tumor tissue and resulted in decreased diversity of the TCR repertoire. Diversity of TCRs expressed by T_reg_ cells was highest in control lymph nodes, not subject to antigen stimulation (consistent with the analysis of unmanipulated mice), and decreased in tumor draining lymph nodes and in tumors. This suggests that T_reg_ cells are subject to the most prominent constriction of the TCR repertoire and further implies that many TCRs expressed in control lymph nodes (and in healthy mice) will not be present in tumors (Fig. 4A). Analysis of the TCR repertoire overlap was conducted to reveal relationship between T cell populations. This analysis shows that T_reg_ cells in control lymph nodes, that mostly resemble T_reg_ population in unmanipulated mice, express most divergent TCR repertoire from all other T cell subsets. In contrast, T_reg_ cells in tumors and tumor draining lymph nodes are more similar to activated T cell subsets from the respective anatomic locations. This suggests that these T_reg_ subsets include higher proportion of cells expressing the same TCRs as activated cells. TCR repertoires of activated T cells in control lymph nodes express intermediate TCR repertoire between activated and T_reg_ cells from tumor draining lymph nodes and tumors and naive cells. Both naive cell populations in control and draining lymph nodes are similar, consistent with the lack of antigen mediated selection (Fig. 4B). In conclusions, statistical analysis conducted on TCRs indicates close relationship between activated and T_reg_ cells in tumor draining lymph nodes and tumors, significant divergence of T_reg_ cells in control lymph nodes from all other T cell subsets and close similarity of naive cells in control and tumor draining lymph nodes.

**Figure 5:**
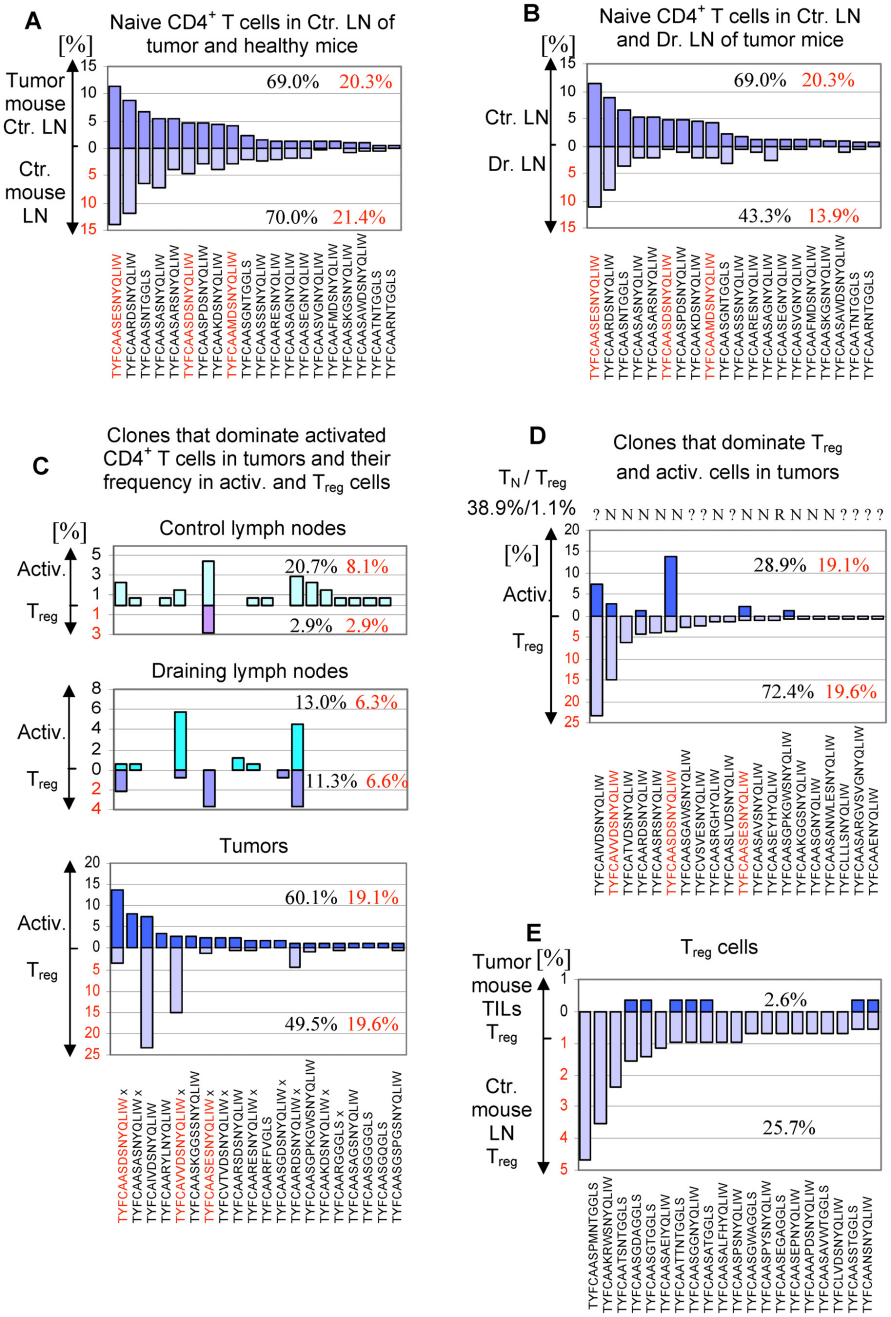
Analysis of the TCR repertoire in tumor-bearing TCR^mini^-Foxp3^GFP^ mice. Sequences of the TCRα chain CDR3 regions are shown below plots, red sequences mark T cell clones specific for Ep63K peptide. Numbers indicate the percentage of clones shown on the plot (black) and the percentage of the Ep63K-specific cells (red) in the total population of the T cell subset analyzed. (A) Naive CD4^+^ T cells in healthy and tumor-bearing TCR^mini^-Foxp3^GFP^ mice express similar TCR repertoires. Frequencies (%) of the 20 most abundant clones in control lymph nodes of mice with tumors (dark purple) and in lymph nodes of healthy mice (light purple) are shown. (B) Comparison of TCR repertoires of naive CD4^+^ T cells in the control (dark purple) and the draining lymph nodes (light purple) of TCR^mini^-Foxp3^GFP^ mice with tumors. 20 most abundant clones in the control lymph nodes is shown. (C) Analysis of frequency of activated (Activ.) and T_reg_ cell clones in control (upper panel) and draining (middle panel) lymph nodes and tumors (lower panel). 20 most abundant clones in the population of activated T cells in tumors was selected for analysis. TCRs marked with “x” were also found in B16 tumors not expressing Ep63K epitope. (D) The abundance of T cell clones expressing TCRs shared with naive/effector CD4^+^ T cells (N) or expressing TCRs exclusive for the T_reg_ subset (R) in the populations of activated (blue, upper part of the panel) and T_reg_ (purple, lower part of the panel) cells in tumors. Clones selected for analysis are the 20 most abundant clones in the population of T_reg_ cells in tumors. Receptors found in naive/effector T cells (N) and Treg cells (R) are shown above the plot. Receptors not assigned to any subset are labeled “?”. (E) The frequency of T_reg_ clones (blue) expressing the exclusive set of TCRs in tumors. Clones selected for analysis are the 20 most abundant clones in the population of T_reg_ cells in healthy mice (purple).

To examine cellular processes in cancer immune response, we followed individual T cell clones in populations of naive, activated and T_reg_ cells in control and draining lymph nodes and in tumors. Naive cells in control lymph nodes were least affected by the ongoing anti-tumor response. Single-cell analysis shows a remarkable consistency between TCR repertoires of individual TCR^mini^-Foxp3^GFP^ mice. Of the 20 most abundant clones in the population of naive CD4^+^ T cells in control lymph nodes of tumor-bearing mice, 16 were also most abundant in naive cells of unmanipulated mice (Fig. 5A). T cells expressing these receptors constituted 69.0 and 70.0% of the respective naive populations. Similar comparison between the 20 most abundant clones of naive cells in control and the draining lymph nodes of tumor-bearing mice shows that only 10 clones were the same in the later population and they amounted to only 43.3% of all naive cells (Fig. 5B). Differences in the TCR repertoires of naive T cells in control and draining lymph nodes are most likely caused by recruitment of antigen-specific naive cells into draining lymph nodes before they acquire activation markers.

**Figure 6:**
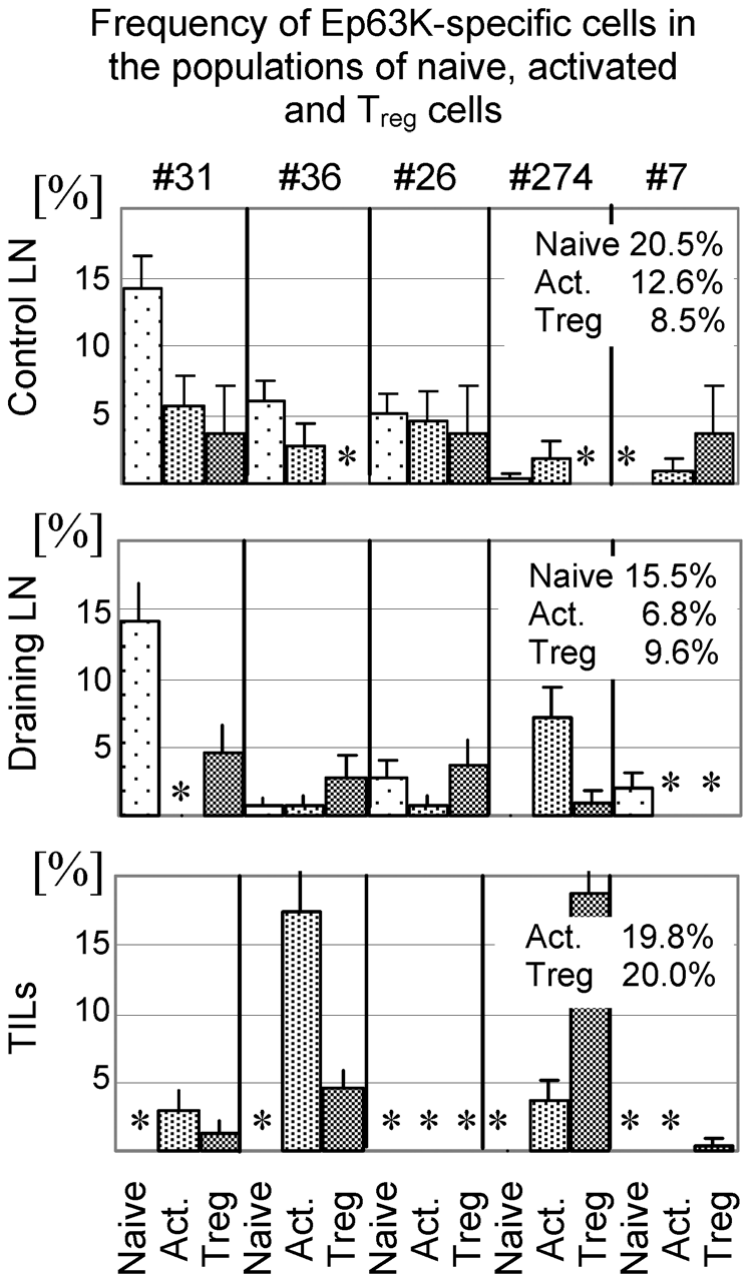
Clonal abundance (%) of Ep63K-specific T cell clones in naive (sparse dots), activated (dense dots) and T_reg_ (stripes) subsets in the control and draining lymph nodes and tumors of tumor-bearing TCR^mini^-Foxp3^GFP^ mice. The percentage of all Ep63K-specific clones in populations of naive, activated and T_reg_ cells is shown on each plot. Names of hybridomas expressing a particular TCRα chain are shown above upper panel. Asterisks indicate that no clones were found in the indicated cell subsets.

In contrast to the population of naive cells, TCR repertoires of activated cells show more pronounced differences between anatomical locations. Analysis of the twenty most abundant T cell clones in tumor tissue shows that they account for about 60.0% of activated TILs (Fig. 5C). Six of these clones were also found in the tumor draining lymph nodes (13.0% of activated T cells) and fourteen were found in control lymph nodes (20.7% of activated cells). Thus, not all T cell clones predominating in the tumors are also present in the tumor draining lymph nodes suggesting that some tumor-specific T cells may migrate directly to tumors. The same T cell clones accounted for 2.9% of all T_reg_ cells in the control lymph nodes, 11.0% of T_reg_ cells in the draining lymph nodes and 49.5% of T_reg_ cells in the tumor tissue. This shows increased contribution of effector T cells to the population of T_reg_ cells. This process is well exemplified by Ep63K-specific T cells (Fig. 6). The distribution of cells between the populations of activated and T_reg_ cells varied for individual Ep63K-specific T cell clones. Three clones (#26, 31, 36) frequently found in control lymph nodes were also found in the draining lymph nodes, however we did not observe their clonal expansion. These clones were also found abundant in unmanipulated mice and did not expand upon immunization with Ep63K-peptide and CFA [20]. Only one of these clones (#36) was found expanded in tumors mainly in the population of activated T cells. While two clones dominate immune response to Ep63K peptide and CFA (#274 and 7), in tumor-bearing mice only one clone (#274) was abundant in B16 tumors expressing NP-Ep63K [20]. This might be caused by the differences in the conformation of Ep63K epitope when it is presented as a soluble peptide or processed from NP-Ep63K protein. Since clone #274 was also found in B16 tumors not expressing Ep63K (see below), it is likely that its expansion in modified tumors resulted from the recognition of both Ep63K and a natural, unknown epitope produced by melanoma cells. Clone #274 constituted 5.7 and 0.7% of activated and T_reg_ cells in draining lymph nodes and 2.9 and 14.9% of activated and T_reg_ cells in tumors. Thus, this clone is a good example of a major functional change experienced by effector CD4^+^ T cells and while it constituted about 90.0% of activated and 10.0% of Ep63K-specific T_reg_ cells in the draining lymph nodes, in tumors it accounted for only 15.0% of activated and 76.0% of Ep63K-specific T_reg_ cells. In summary, TCR repertoire analysis suggests efficient recruitment of tumor-antigen specific cells into tumor lesions and tumor draining lymph nodes. The initial encounter with tumor derived antigens most likely occurs in the tumor-draining lymph nodes where specific clones were first found expanded. Further expansion occurring in the tumor is associated with induction of the Foxp3 expression and acquisition of the suppressor phenotype. Increased contribution of Ep63K-specific clones to the population of T_reg_ cells in tumors is paralleled by other CD4^+^ T cell clones dominating the population of activated CD4^+^ T cells in tumors. These clones constitute 41.0, 6.7 and 12.6% of activated CD4^+^ T cells and 29.9, 4.7 and 0% of T_reg_ cells in tumors, tumor draining lymph nodes and control lymph nodes respectively. Ten TCRs (marked with x) of the twenty that dominate the population of activated TILs were also found in the population of CD4^+^CD25^+^ cells isolated from B16 tumors not expressing Ep63K epitope (Fig. 5C). Due to limited sampling, the TCRs we found in B16 tumors expressing and devoid of Ep63K likely represent only partial overlap of the TCR repertoires. Thus, analysis of the TCR repertoires of activated and T_reg_ cells in various anatomic locations shows that both tumor neo-antigen and natural B16-associated antigens shape the repertoires of CD4^+^ T cell subsets. In conclusion, activation/expansion of effector CD4^+^ T cells is associated with the increased proportion of T_reg_ cells expressing TCRs shared with effector cells in tumors.

We have established that T_reg_ cells expressing TCRs shared with effector CD4^+^ T cells constitute about half of all T_reg_ cells in the tumor site while in unmanipulated mice they account for only 10-15% of all T_reg_ cells (Fig. 5C)[20]. Most T_reg_ cells in normal mice, not subjected to antigen stimulation express an exclusive set of TCRs, different from TCRs expressed by effector T cells, so it was important to determine the contribution of this subset to the T_reg_ population in tumors. We have selected twenty T_reg_ clones most abundant in tumors (account for 72.4% of all T_reg_ cells) and using our database of TCR sequences collected from unmanipulated and tumor-bearing mice identified clones that express TCRs shared with naive T cells and TCRs unique for the T_reg_ cells (Fig. 5D). We found that eleven TCRs expressed by 38.9% of all T_reg_ cells were also expressed by naive cells and only one TCR (expressed by 1.1% of T_reg_ cells) was found solely in T_reg_ subset. Other receptors were not found in the database. The same analysis conducted on eight T_reg_ clones, with unknown antigen specificity, shows that TCRs shared with effector CD4^+^ T cells (receptor sequences depicted in black and labeled with N) constitute 18.2% and 3.7% of all T_reg_ cells in tumors and tumor draining lymph nodes respectively and are not found in the control lymph nodes. In summary, T_reg_ cells expressing TCRs shared with effector cells constitute an increasing proportion of all T_reg_ cells and contribute to the TCR diversity of these cells.

To further investigate composition of T_reg_ subsets in tumors, we looked at T_reg_ clones expressing twenty most frequent TCRs exclusive for T_reg_ cells (Fig. 5E). This set of TCRs accounted for 25.7% of all T_reg_ cells in lymph nodes of unmanipulated mice, 26.0% of T_reg_ cells in the draining lymph nodes of tumor-bearing mice (10 TCRs found) and for only 2.6% of all T_reg_ cells in tumors (7 of these TCRs were found). Extending this analysis to all T_reg_-specific TCRs, we found that of 79 unique TCRs found in tumor-draining lymph nodes 48 TCRs belonged to a TCR subset found exclusively in T_reg_ cells while of 91 unique TCRs found in tumors only 2 TCRs belonged to this cell subset despite sequencing twice more receptors from tumor tissue. This surprising result suggests that T_reg_ cells expressing a unique set of TCRs and predominating in normal mice have different behavior than T_reg_ cells expressing TCRs shared with effector T cells present in tumor-bearing mice. In addition, the most abundant T_reg_ clone expressing a TCR found only in T_reg_ cells accounted for 1.1% of all T_reg_ cells in tumors. While we do not know why T_reg_ cells expressing an exclusive set of TCRs are underrepresented in the tumor infiltrate with regard to both clonal abundance and TCR diversity, possible reasons include impaired migration to tumors, inferior expansion and/or very low frequency of these cells in mice before tumor inoculation. In summary, TCR repertoires of T_reg_ cells in tumor draining lymph nodes and tumors, sites critical for successful tumor eradication, is progressively dominated by T_reg_ cells expressing the same TCRs as effector T cells. In contrast, T_reg_ cells expressing an exclusive set of TCRs that dominate TCR repertoires of T_reg_ cells in unmanipulated mice, and even in the draining lymph nodes of tumor bearing mice constitute disproportionally small fraction of T_reg_ cells in tumors.

### Inherent properties of the T_reg_ subsets expressing TCR shared with effector CD4^+^ T cells are responsible for their dominance in the T_reg_ population in tumors

**Figure 7:**
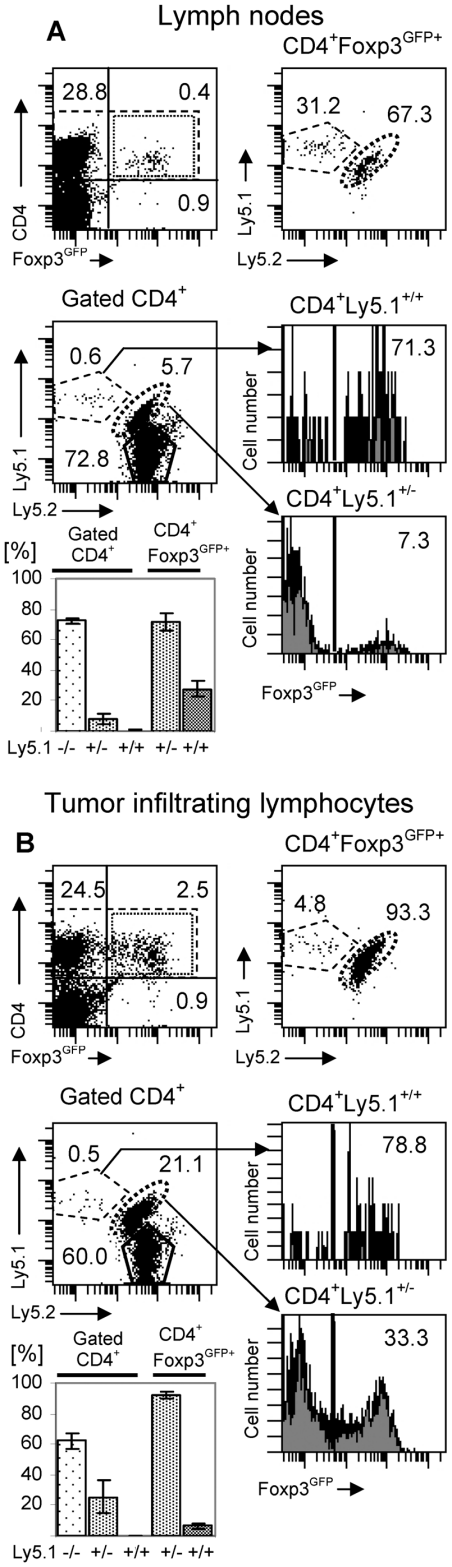
Adaptive T_reg_ cells expressing wild-type TCR repertoire dominate T_reg_ cell population in melanoma tumors. TCR^mini^ mice were inoculated with B16 melanoma tumors and, after 3 days, reconstituted with CD4^+^Foxp3^GFP-^ from Ly5.1^+/−^Foxp3^GFP^ mice or CD4^+^Foxp3^GFPhi^ cells from Ly5.1^+/+^Foxp3^GFP^ mice. Lymph nodes (A) and tumor infiltrating lymphocytes (B) were analyzed by flow cytometry 12 days after cell transfer. (A) Flow cytometry analysis of the CD4^+^ T cell population in tumor draining lymph nodes. Upper left dot plot shows Foxp3^GFP^ expression (dotted line rectangle) in CD4^+^ T cells (dashed line rectangle). Numbers represent proportions of cells in each quadrant. Lower left dot plot shows proportions of recipient Ly5.1^−/−^ T cells and donor Ly5.1^+/+^Foxp3^GFPhi^ and Ly5.1^+/−^Foxp3^GFP-^ cells in the total population of gated CD4^+^ T cells. Gate used to define CD4^+^ T cells is shown by dashed rectangle on the upper left plot. Proportions of cells expressing Foxp3^GFP^ in donor CD4^+^Ly5.1^+/+^ and CD4^+^Ly5.1^+/−^ cells are shown on upper and lower histograms respectively. Upper right dot plot shows proportions of lymph node CD4^+^Foxp3^GFP+^ T cells generated by donor Ly5.1^+/+^Foxp3^GFPhi^ and Ly5.1^+/−^Foxp3^GFP-^ cells. Gate used to define CD4^+^Foxp3^GFP+^ T cells is shown by dotted rectangle on the upper left dot plot. The data shown is representative of three mice analyzed. The summary of the data for gated total CD4^+^ cells and CD4^+^Foxp3^GFP+^ cells is presented on the bar graph. (B) Flow cytometry analysis of the CD4^+^ T cell population in tumors. Lymphoid cells were purified on Lympholyte-M gradient. All dot plots and histograms show the same cell subsets as analyzed in the lymph nodes.

Analysis of the clonal abundance of T_reg_ cells in TCR^mini^-Foxp3^GFP^ mice bearing B16 melanoma tumors expressing a neoantigen led to the conclusion that T_reg_ cells in tumors are dominated by Foxp3^GFP+^ cells expressing TCRs shared with effector T cells. To complement our experiments, we have investigated the origin of T_reg_ cell populations expressing a wild-type diversity of TCRs and generated in B16 tumors not expressing a neoantigen. CD4^+^Foxp3^GFP-^ and CD4^+^Foxp3^GFPhi^ cells that could be distinguished by allelic markers Ly5.1^+/−^ and Ly5.1^+/+^ were sorted by flow cytometry. Sorted conventional and T_reg_ populations, expressing reciprocal allelic markers, were mixed and adaptively transferred into TCR^mini^Ly5.1^−/−^ mice (not expressing Foxp3^GFP^ transgene) that were inoculated with B16 melanoma tumors (not expressing a neoantigen). CD4^+^Foxp3^GFPhi^ cells, that constituted 13% of all transferred T cells, express mostly TCRs found almost exclusively in the T_reg_ population. Since TCR^mini^ mice are not lymphopenic, transferred cells do not undergo homeostatic expansion leading to spontaneous upregulation of Foxp3 and CD4^+^Foxp3^GFP-^ cells that acquire Foxp3 expression represent adaptive T_reg_ cells generated in response to self peptides or antigens derived from commensal flora [20], [41]. Figure 7 shows analysis of recipient mice that received populations of CD4^+^Ly5.1^+/−^Foxp3^GFP-^ and CD4^+^ Ly5.1^+/+^Foxp3^GFPhi^ cells. Similar result was obtained when conventional and T_reg_ cells expressing, respectively, Ly5.1^+/+^ and Ly5.1^+/−^ allelic markers were transferred. CD4^+^Foxp3^GFPhi^ cells represented 9.5% of transferred cells and accounted for 31.2% of all T_reg_ cells in lymph nodes of recipient mice (Fig. 7A). However, the same cells in the tumors represented only 2.3% of all transferred cells and 4.8% of T_reg_ cells (Fig. 7B). This low proportion of T_reg_ cells in tumors originating from the preexisting Foxp3^+^ cells is a consequence of superior recruitment/expansion of CD4^+^Foxp3^GFP-^ cells in the tumor tissue with simultaneous increase in the proportion of Foxp3^GFP+^ T cells in this subset. This outcome is consistent with recent reports that the population of T_reg_ cells exhibiting a flexible phenotype and expressing TCRs shared with effector cells (and representing adaptive T_reg_ cells) expands much better in response to antigen stimulation or in lymphopenic mice than T_reg_ cells expressing an exclusive set of TCRs that are found in Foxp3^GFPhi^ population sorted from unmanipulated mice [20], [23]. Increased proportion of T_reg_ cells in melanoma tumors than in peripheral blood and peritumoral tissue was also reported in melanoma patients and was attributed to selective migration and/or expansion of T_reg_ cells in tumors [42]. Both Ep63K-specific CD4^+^ T cells and effector cells with unknown antigen specificity present in the TCR^mini^ mice as well as adaptively transferred CD4^+^Foxp3^GFP-^ cells expressing a wild type diversity of the TCR repertoire were found dominating the population of T_reg_ cells in melanoma tumors. In conclusion, inherent properties of T_reg_ cells expressing TCRs shared with naive T cells like efficient recruitment, expansion and/or upregulation of Foxp3 are responsible for the predominance of adaptive T_reg_ cells in tumors.

### Immunotherapy with dendritic cells presenting tumor-associated antigen fails to prevent generation of adaptive T_reg_ cells in tumor tissue

**Figure 8:**
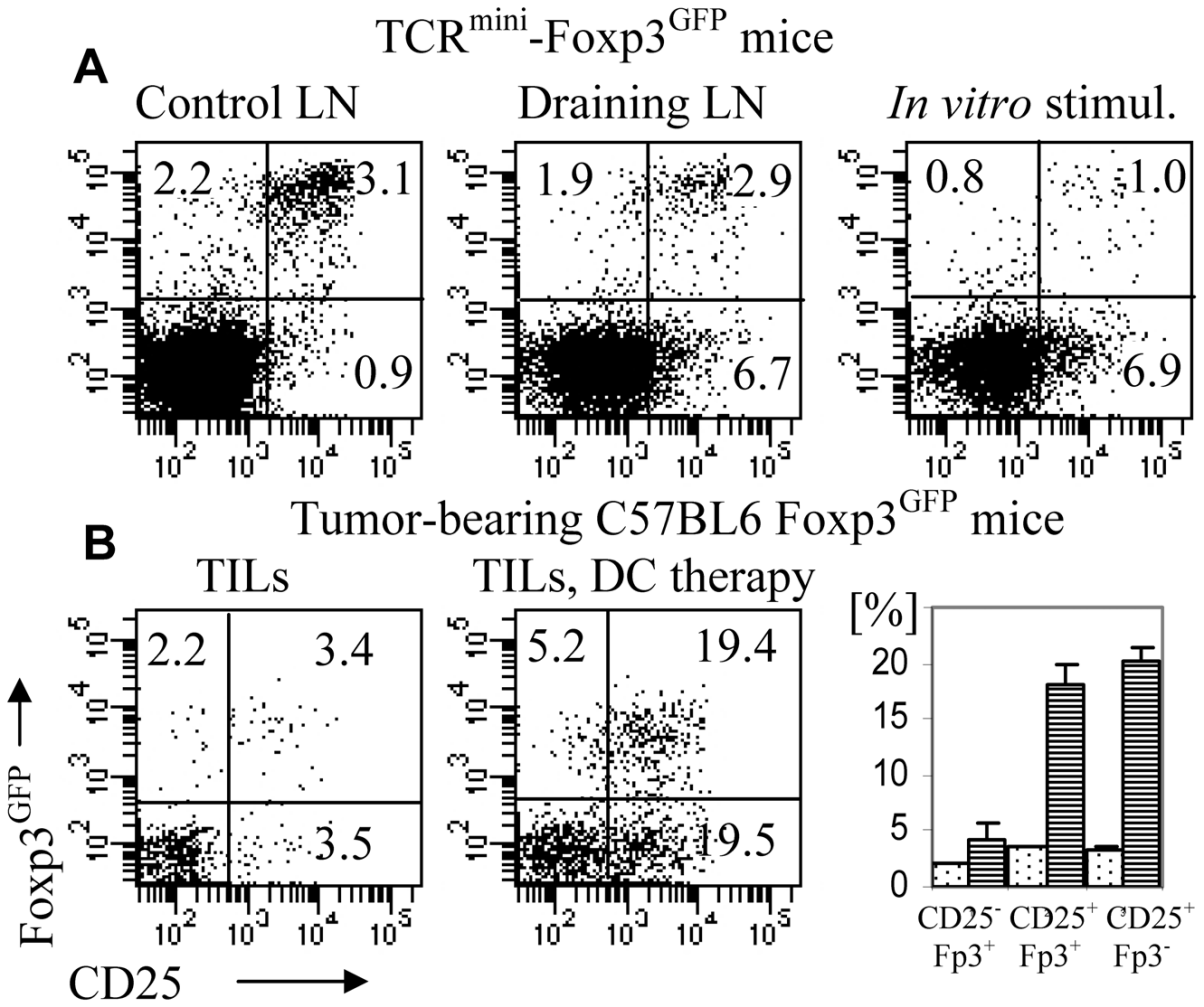
Flow cytometry analysis of CD4^+^ T cells stimulated with dendritic cells presenting Ep63K peptide. Expression of Foxp3^GFP^ and CD25 is shown on gated CD4^+^ T cells. (A) Analysis of the CD4^+^ T cells from control (left) and draining (middle) lymph nodes of TCR^mini^-Foxp3^GFP^ mice immunized with dendritic cells presenting Ep63K and CD4^+^ T cells isolated from TCR^mini^-Foxp3^GFP^ mice and stimulated with dendritic cells *in vitro* (right). Dendritic cells (2×10^5^) were injected into footpads of TCR^mini^-Foxp3^GFP^ mice for three days and mice were sacrificed after 5 days and popliteal lymph nodes were analyzed. (B) Analysis of gated CD4^+^ T cells isolated from tumors of tumor-bearing TCR^mini^-Foxp3^GFP^ control mice (left) and mice immunized with dendritic cells (middle). TCR^mini^-Foxp3^GFP^ mice were inoculated with B16 melanoma cells expressing NP-EP63K and at the same time dendritic cells were injected (5×10^4^). Injections of dendritic cells continued daily until mice were sacrificed. The abundance (%) of CD4^+^ T cells subsets CD25^-^Foxp^GFP+^, CD25^+^Foxp^GFP+^ and CD25^+^Foxp^GFP-^ in the tumor tissue of TCR^mini^-Foxp3^GFP^ control (dots) and immunized (stripes) mice. At least three mice were analyzed in each group.

We reasoned that the overwhelming presence of adaptive T_reg_ cells in tumors might be caused by suboptimal antigen stimulation in tumors compared to draining lymph nodes and could be corrected by immunotherapy. Previous reports showed that stimulation with limited antigen dose and premature termination of TCR signaling promotes generation of T_reg_ cells [43], [44]. To improve the ratio of effector and T_reg_ cells, we augmented immune response to tumor-associated antigen using dendritic cells. C57BL6 Foxp3^GFP^ mice (expressing wild type TCR repertoire) were inoculated with B16 melanoma expressing NP-Ep63K. Bone marrow-derived dendritic cells expressing covalently bound complex of A^b^Ep63K complex were co-injected in the same site at the time of tumor inoculation [33]. Injections of dendritic cell continued daily until mice were sacrificed. Dendritic cells expressing A^b^Ep63K complex were devoid of wild type A^b^ molecule and also lacked invariant chain [45]. Invariant chain deficiency prevents cleavage of the Ep63K peptide from A^b^ and ensures that all class II MHC present one, covalently bound peptide. This excludes the possibility that some other class II MHC associated peptides could be recognized by CD4^+^ T cells and result in T_reg_ induction and ensures that both effector and T_reg_ cells are stimulated against the same antigenic peptide produced solely by tumor cells. Dendritic cells expressing A^b^Ep63K complex were able to stimulate Ep63K-specific CD4^+^ T cells isolated from TCR^mini^-Foxp3^GFP^ mice *in vitro* or *in vivo* without significant increase in the proportion of T_reg_ cells (Fig. 8A). Tumor-bearing mice were sacrificed after two weeks and T cells from control and draining lymph nodes and tumor tissue were analyzed by flow cytometry (Fig. 8B). Mice undergoing dendritic cell therapy had more CD4^+^ T cells in tumor-draining lymph nodes and tumors and a higher fraction of CD4^+^ T cells expressed an activated phenotype what shows that dendritic cells were effectively stimulating Ep63K-specific T cells. However, augmented activation of effector CD4^+^ T cells was accompanied by the concomitant increase in the fraction of T_reg_ cells. In conclusion, stimulation of tumor antigen-specific effector cells is associated with conversion and/or expansion of T_reg_ cells specific for the same antigen. This finding suggests that efficient presentation of antigen might not be sufficient to favor the generation of activated T cells in the context of tumor microenvironment.

## Discussion

Emerging data suggest that the population of Foxp3^+^ T_reg_ cells is heterogeneous in humans and mice in terms of its origin and functional properties [19], [20], [24], [26]. However, since the expression of the cell surface markers may change reflecting different activation or functional status of a T_reg_ cell, it might be difficult to follow T_reg_ subsets in the course of immune response. In a recent report we were able to correlate functional phenotype of T_reg_ cells with the expression of a TCR [20]. Thus, we could use the analysis of the TCR repertoire in TCR^mini^-Foxp3^GFP^ mice to determine the origin and abundance of T_reg_ subsets expressing TCRs shared with effector T cells and expressing an exclusive set of TCRs.

Despite recent advances in immunotherapy, T_reg_ cells remain the major obstacle for successful cancer treatment. It is therefore important to determine what is the source and cellular composition of T_reg_ cells in tumors. We investigated T_reg_ population in tumor-bearing TCR^mini^-Foxp3^GFP^ mice expressing naturally rearranged, polyclonal TCR repertoire. To our knowledge this is the first model that allows for comprehensive assessment how the origin and antigenic specificity of T cell populations change in the course of tumor development. Considering the major impact of T_reg_ cells on cancer pathology, we have investigated the origin of these cells, anatomical location where tumor specific T_reg_ cells are first detectable and the stage of tumor growth when they are first expanded.

To follow tumor antigen-specific CD4^+^ T cells, B16 melanoma was modified to express NP-Ep63K recombinant protein. Processing of the recombinant protein by antigen presenting cells generates Ep63K epitope that mimics tumor associated antigens that arise from mutant, endogenous proteins for which immune system is not tolerant but which were proposed to be the most efficient targets for immunotherapy [37], [38], [46]. The inclusion of an experimental neoantigen was dictated by multiple reports showing that tumors naturally produce antigenic epitopes derived from mutant as well as native proteins. These antigenic epitopes are able to elicit activation, clonal expansion and acquisition of effector functions by antigen specific cytotoxic and helper CD4^+^ T cells [47]–[51]. In conclusion, by using tumor cells expressing a neoantigen, we facilitate studies how cellular mechanisms are established that protect tumors from the immune system and result in the induction of an active tolerance/anergy mediated by T_reg_ cells [14], [52].

Analysis of the TCR repertoires in TCR^mini^-Foxp3^GFP^ mice bearing B16 tumors expressing NP-Ep63K demonstrates that the population of T_reg_ cells expressing TCRs shared with naive CD4^+^ T cells, and not the T_reg_ cells expressing an exclusive set of TCRs, constitute a majority of Foxp3^+^ T_reg_ cells in tumors. The recent report and our own data show that the population of peripheral Foxp3^+^ T_reg_ cells in unmanipulated mice is heterogeneous and includes a subset expressing TCRs shared with effector T cells [20], [23]. Thus, it is not possible to demonstrate whether an individual T_reg_ cell expressing a shared TCR in tumor-bearing mice arose from expansion of a cell preexisting in a healthy mouse, or from an effector T cell converted during tumor antigen-driven response and thus representing an adaptive T_reg_ cell. This distinction may not be very meaningful since we have recently showed that T_reg_ cells in healthy mice expressing shared receptors resemble adaptive T_reg_ cells [20]. At the population level, most likely, both expansion of the preexisting T_reg_ cells expressing shared TCRs and antigen-driven conversion contribute to the abundance of the T_reg_ subset expressing TCRs shared with effector cells. Considering the large size of the population of naive CD4^+^ T cells and a comparably small proportion of T_reg_ cells expressing shared TCRs, most of T_reg_ cells in tumors are likely generated by conversion of effector CD4^+^ T cells. This view is supported by evidence of efficient expansion of the preexisting adaptive T_reg_ cells in immunized mice, adaptive transfer studies and by published report demonstrating that conversion into T_reg_ cells is an efficient mechanism of acquiring Foxp3 expression [53], [54].

Conversion and/or expansion of the T_reg_ cells expressing shared TCRs might be a general feature of response to antigen since these cells were also demonstrated in the draining lymph nodes of mice immunized with peptide antigen and CFA [20]. Thus, regardless of the origin of antigen and possible differences in the mode of antigen presentation, adaptive T_reg_ cells are generated both in immunized and tumor-bearing mice. Inflammation caused by immunization with CFA did not prevent the generation of adaptive T_reg_ cells. In fact, inflammation was found to be a contributing factor in many cancers and our data imply that cancer treatments aimed at stimulating inflammatory reaction in the site of tumor growth may not promote tumor eradication [55].

Analysis of the frequency of individual Ep63K-specific clones reveals that the magnitude of clonal expansion might correlate with the proportion of T cells that acquire Foxp3 expression and become T_reg_ cells. The major increase in the abundance of antigen-specific cells occurred for T cells expressing TCRs of hybridoma #274 that is rarely found in unmanipulated mice but dominates antigen response to conventional immunization with peptide antigen and CFA. Another Ep63K-specific clone expressing TCR of hybridoma #36 was found modestly expanded in tumors though it was not expanded upon immunization with Ep63K-peptide and CFA [20]. In contrast, CD4^+^ T cells expressing TCRs utilized by hybridomas #31 and 26 were not expanded in tumor draining lymph nodes and tumors. This is most likely caused by their low affinity for Ep63K since the same cells also did not respond to immunization with soluble Ep63K administered with CFA. Analysis of clonal frequencies in tumors shows that clone #274, that expanded the most, had highest contribution to the T_reg_ population, clone #36, that expanded less, had smaller contribution and clones #31 and #26, that did not expand, were rarely seen in activated and T_reg_ cells in tumors. The association between antigen-driven clonal expansion and acquisition of T_reg_ phenotype was seen in cervical cancer patients and in tumor-bearing mice following vaccination suggesting that it is likely a general phenomenon associated with antigen stimulation [56], [57]. However, previous studies in a mouse model have relied on TCR-transgenic T cells that may not reflect the behavior of a naturally-diverse polyclonal TCR repertoire.

Ep63K-specific CD4^+^ T cells undergo efficient clonal expansion in response to antigen produced by tumor cells. This experimental system mimics the desired response of the immune system to tumor associated antigen. Some natural tumor antigens, produced at lower levels or less immunogenic, may not elicit efficient cellular response. Our data strongly suggest that immunotherapy targeting this type of antigens by augmenting their antigenicity will have a similar outcome as the response to a highly immunogenic antigens. Activation and clonal expansion of effector CD4^+^ T cells will result in the generation of adaptive T_reg_ cells and/or expansion of T_reg_ cells sharing TCR with effector T cells.

The small contribution of T_reg_ cells (both in terms of TCR diversity and clonal abundance) expressing an exclusive set of TCRs and preexisting in healthy mice to the population of Foxp3^+^ T cells in tumors is surprising, especially considering that these cells still constitute a significant proportion of Foxp3^+^ T_reg_ cells in the tumor draining lymph nodes. We do not think it is an artifact of our experimental approach. Our conclusions are based on the analysis of single cells that avoids bias that might be introduced by sequencing TCRs from the library amplified by the PCR. In addition, analysis of the clonal abundance accounts for over 70% of all Foxp3^+^ T cells in tumor tissues so it is unlikely that we missed some T_reg_ clones that account for a large fraction of Foxp3^+^ cells in tumors. T_reg_ cells in unmanipulated mice (expressing mainly an exclusive set of TCRs) consistently had the most diverse TCR repertoire in several unmanipulated mice analyzed so far, so it is unlikely that they do not contain tumor-antigen specific cells. We hypothesize that migration or tissue homing of these cells may be different from the majority of T_reg_ cells found in tumors. This interpretation is further supported by the demonstration that T_reg_ cells expressing an exclusive set of TCRs constitute a significant fraction of T_reg_ cells in the tumor draining lymph nodes and by the adaptive transfer experiment showing that T_reg_ cells derived from transferred effector cells are more efficiently recruited and expanded in the tumors than T_reg_ cells preexisting in unmanipulated mice. In fact, recent transcriptional analysis of tumor-derived T_reg_ cells shows that they express a different gene expression profile than T_reg_ cells in healthy mice [58].

Analysis of early and late stage tumors shows that composition of the cellular infiltrate does not change with tumor progression. In contrast, our data show that, at least for the widely used transplantable tumor model, cellular changes that compromise the functions of the immune system do not occur following productive immune response but are evident already at the stage when tumors are first detectable. Tumor models that progress through the salient period of preclinical cancer are needed to investigate how immune response evolves in more natural cancer models but some preliminary data indicate that it may also be compromised at the very early stages of cancer [59]. In fact, recent report demonstrates that T_reg_ cells expand dynamically upon tumor antigen recognition and create tolerogenic environment in tumors from the onset of tumor growth [60]. Though, the analysis of the TCR repertoire of expanded T_reg_ cells was not conducted in this study, it was shown that at least some T_reg_ cells expressed the same TCRs as effector T cells.

Our analysis of the immune response in cancer and previously reported analysis of immune response to peptide antigen administered with strong adjuvant reveals several common features [20]. In both cases antigen-specific effector T cells are efficiently recruited into draining lymph nodes and undergo clonal expansion. Thus, impaired clonal expansion or lack of it is an unlikely cause of the failed response in cancer. Another common feature is that T_reg_ population becomes enriched in cells expressing TCRs shared with effector cells, either by upregulation of Foxp3 in effector T cells, or by expansion of a minor T_reg_ subset. Generation of T_reg_ cells that share TCRs with effector T cells is not a result of suboptimal stimulation by antigen. In contrast, augmenting immunization with professional antigen presenting cells increases generation of both antigen-specific effector and adaptive T_reg_ cells.

In our model, we show that some adaptive T_reg_ cells can be generated in the course of a productive immune response, against antigens presented via conventional immunization. This process enriches T_reg_ population in cells with the same antigen specificity as effector cells and would be consistent with the known role of induced T_reg_ responses in limiting excessive inflammation and tissue damage [61]. In the case of tumors, it appears that the natural mechanisms existing to generate or expand T_reg_ cells during response to conventional antigen become exaggerated and subverted by tumor cells. This elicits disproportionate production of T_reg_ cells and shifts the cellular balance from effector helper cells towards cells with suppressor function in order to avoid immune system mediated destruction. The outcome of immunization with dendritic cells expressing the same antigen as tumor cells demonstrates that even strong stimulation with antigen in the tumor site still does not shift the balance to favor production of effector T cells at the expense of T_reg_ cells. This further suggests that improved cancer immunotherapy may depend on the ability to block tumor-antigen induced expansion of a minor T_reg_ subset or generation of adaptive T_reg_ cells, rather than solely on increasing the immunogenicity of vaccines.
